# Supplementary material for: Predicting suitable habitat for the endangered tree Ormosia microphylla in China
Source: Sci Rep. 2024 May 6;14:10330. doi: 10.1038/s41598-024-61200-5 (PMC11074134; doi:10.1038/s41598-024-61200-5)
Supplement: Supplementary file 1 — Supplementary Information. [file 41598_2024_61200_MOESM1_ESM.docx]

Predicting suitable habitat for the endangered tree *Ormosia microphylla* in China

Lijuan Wei ^1,†^, Guohai Wang ^2,†^, Chunping Xie ^3,^*, Zequn Gao ^2^, Qinying Huang ^2^ and C. Y. Jim^4,^*

^1^ College of Mathematics, Physics and Electronic Information Engineering, Guangxi MinZu Normal University, Chongzuo 532200, China

^2^ College of Chemistry and Bioengineering, Guangxi MinZu Normal University, Chongzuo 532200, China;

^3^ Tropical Biodiversity and Bioresource Utilization Laboratory, Qiongtai Normal University, Haikou 571127, China

^4^ Department of Social Sciences and Policy Studies, Education University of Hong Kong, Tai Po, Hong Kong, China

***** Correspondence: xcp@mail.qtnu.edu.cn; cyjim@eduhk.hk

^†^ These authors contributed equally to this work.

**Abstract**

Climate change has significantly influenced the growth and distribution of plant species, particularly those with a narrow ecological niche. Understanding climate change impacts on the distribution and spatial pattern of endangered species can improve conservation strategies. The MaxEnt model is widely applied to predict species distribution and environmental tolerance based on occurrence data. This study investigated the suitable habitats of the endangered *Ormosia microphylla* in China and evaluated the importance of bioclimatic factors in shaping its distribution. Occurrence data and environmental variables were gleaned to construct the MaxEnt model, and the resulting suitable habitat maps were evaluated for accuracy. The results showed that the MaxEnt model had an excellent simulation quality (AUC=0.962). The major environmental factors predicting the current distribution of *O. microphylla* were the mean diurnal range (bio2) and precipitation of the driest month (bio14). The current core potential distribution areas were concentrated in Guangxi, Fujian, Guizhou, Guangdong, and Hunan provinces in south China, demonstrating significant differences in their distribution areas. Our findings contribute to developing effective conservation and management measures for *O. microphylla*, addressing the critical need for reliable prediction of unfavorable impacts on the potential suitable habitats of the endangered species.

**Keywords:** *Ormosia microphylla*; climate change; MaxEnt model; suitable area; species distribution model (SDM); conservation measure

1. Introduction

Climate change has influenced the growth and survival of plant species, especially those with a narrow ecological niche [1](#_ENREF_1)^,^[2](#_ENREF_2). Extreme high temperatures and droughts, incurring abiotic and biotic stresses, can harm plant growth and forest health [3](#_ENREF_3). Moreover, changes in environmental conditions could alter physiological responses, metabolic processes, phenology, and seed dispersal. Plants may respond by adapting to the new conditions or shifting or shrinking their geographical range [4](#_ENREF_4)^,^[5](#_ENREF_5).

Consequently, climate change has brought far-reaching impacts on the distribution patterns of plant species in recent decades, constituting a primary cause of their decline and loss [6](#_ENREF_6). Temperature and precipitation changes brought by climate change modify plant physiological processes, affecting growth, development, reproduction, stability, and geographical distribution [7](#_ENREF_7)^,^[8](#_ENREF_8). Notable and fast climate change can induce serious degradation or loss of species habitats. Plants with weak adaptability and poor dispersal could be driven to local extinction [9](#_ENREF_9). Therefore, assessing climate change impacts on the distribution area and spatial pattern of threatened and endangered plants is crucial for monitoring and restoring native populations in their natural habitats. The results can enhance the formulation of sustainable conservation and management strategies to maintain habitat integrity [10](#_ENREF_10).

In recent years, species distribution modeling (SDM) (e.g., MaxEnt, Random Forests, Bioclim, and Climex) has emerged as an analytical tool for conservation planning and biodiversity management. It is especially useful in poorly surveyed regions beset by increasing habitat degradation and loss pressure [11](#_ENREF_11)^,^[12](#_ENREF_12). SDMs can integrate species occurrence data with environmental variables to simulate and predict habitats suitable for species growth and map the distribution of potential suitable habitats across space and time [13](#_ENREF_13)^,^[14](#_ENREF_14).

Among various SDM algorithms, the Maximum Entropy (MaxEnt) model combines machine learning and maximum entropy principles to predict the potential distribution areas of species [15](#_ENREF_15)^,^[16](#_ENREF_16). The method’s many advantages have triggered extensive application to species distribution modeling. It can utilize both continuous and categorical data and incorporate interactions between variables. It performs better than similar models in forecasting species distribution with small sample size or presence-only data [5](#_ENREF_5). Additionally, the probability distribution obtained from Maxent has a concise mathematical expression, allowing direct generation of a habitat suitability map [17](#_ENREF_17). Each environmental variable's relative importance (%) can be evaluated using the software’s built-in jackknife test [18](#_ENREF_18). These MaxEnt capabilities provide an effectual way to predict the potential distribution of endangered species, which often have a limited number of observed occurrences and grow in remote areas with terrain and access constraints, making field data collection difficult [19](#_ENREF_19).

*Ormosia microphylla* (Merr. & H. Y. Chen) is a dioecious tree species of the Fabaceae family. It is a first-class national protected plant in China. This evergreen tree can reach 15−20 m in height. The legume fruits ripen from October to November. Each fruit holds 3−4 extremely hard seeds that are brightly colored in shades of red. The seeds (150−180 g/1000 grain) rely on wind dispersal. Importantly, the species has high economic value. The attractive seeds are used in jewelry and handicrafts. The priced wood, strong and heavy with handsome grain, is used for furniture and flooring, constituting the main reason for its extensive logging [20](#_ENREF_20).

The natural distribution range of *O. microphylla* is very narrow. It is restricted to forests at 600−800 m altitude on slopes and foot slopes in central and south China, covering Guizhou, Hunan, Guangxi, Guangdong, and Fujian provinces [21](#_ENREF_21). Moreover, the species is beset by low genetic diversity, poor natural regeneration ability, and low seed germination rate, which have jointly depressed its population growth, density, and distribution. Unfortunately, its high economic value, mainly as a valuable timber source has incurred excessive felling by humans. Consequently, the species has suffered from massive tree losses, a severely fragmented range, and increasing scarcity in the wild [22](#_ENREF_22).

Previous *O. microphylla* studies have focused on its population structure [22](#_ENREF_22), with little attention on geographical distribution and factors influencing its habitat. However, its habitat has been seriously damaged, and the natural distribution area has been drastically reduced due to the acute impacts of climate change and human disturbance. Therefore, it is important to study the potential impacts of climate change on the species to yield information about its distribution range, habitat preference, and population dynamics.

This study aimed to investigate the suitable habitat distribution of *O. microphylla* in China with two research objectives: (1) to predict the current potential spatial distribution; and (2) to identify the key environmental factors highly correlated with *O. microphylla* distribution range. The findings will be useful in developing conservation and management measures for the species.

2. Materials and Methods

2.1 Establishing species occurrence records

Occurrence data of *O. microphylla* in China were obtained from the Chinese Virtual Herbarium (http://www.cvh.ac.cn), Plant Photo Bank of China (http://www.Plantphotophoto.cn), National Specimen Information Infrastructure (http://www.nsii.org.cn/), field surveys, and the published literature. Each occurrence record was scrutinized for data quality and suitability with reference to the model's requirements. Records with unclear latitude and longitude information and duplicated distribution points were discarded. A total of 45 verified distribution points of *O. microphylla* were kept for final analysis to generate the potential distribution based on MaxEnt modeling. The occurrence-point data were stored in a CSV file, sorted by species name, longitude and latitude, and plotted on a map (Figure 1).

2.2 Selecting environmental variables

Data on 19 bioclimatic parameters, including monthly temperature and precipitation, were selected and downloaded from the WorldClim website (http://www.worldclim.org) (Table 1). The comprehensive climatic data from 1950 to 2000 were based on monthly meteorological data harvested from different weather stations worldwide. The raw climatic data were interpolated according to a grid with a 2.5-arcminute resolution to generate the global spatial dataset. The rather general annual temperature and precipitation variables were deleted from the dataset [23](#_ENREF_23)^,^[24](#_ENREF_24). Strong correlation among the environmental variables can lower model accuracy. Therefore, Pearson’s correlation coefficients (*r*) were calculated between variable pairs. Variables reaching |>0.8| were removed to avoid multicollinearity impacts [25](#_ENREF_25). Ultimately, we shortlisted eight variables to construct the MaxEnt model for *O. microphylla* in this study.

To avoid model overfitting, the “ENMeval” software package in R 4.0.2 was employed to optimize MaxEnt model [26](#_ENREF_26). For optimization, regular Multiplier (RM) and Feature Class (FC) were included in MaxEnt. RM could smooth the model and minimize model over-fitting. FC, corresponding to the response type of suitability values to each variable, could determine the potential shape of response curves, including Linear (L), Product (P), Quadratic (Q), Threshold (T), and Hinge (H)[27](#_ENREF_27). In the process of parameter optimization, the RM value ranged from 0.5 to 4.0 with an increment of 0.5. Six combinations of FCs (L, LQ, LQP, LQT, QPT, PHT) were selected. Combined with the 8 values of RM, this resulted in a total of 48 parameter combinations. The 48 parameter combinations were input into ENMeval for comprehensive testing. We used the delta from the Akaike information criterion (AIC), AICc, the difference between training and testing AUC (AUC. DIFF) and the 10% training omission rate (OR10) to evaluate the model's fitting degree and complexity on species distribution [28](#_ENREF_28).

2.3 Modeling species distribution

The eight selected environmental variables and species occurrence records of *O. microphylla* were loaded into MaxEnt 3.3. Then, 75% of the distribution points were randomly selected as testing data to establish the prediction model, and the remaining 25% were used to verify the model’s accuracy. To avoid the instability caused by randomly selected data, the initial operation of the model was repeated ten times. A Jackknife test was applied to measure the relative importance of each variable on the distribution of this species by percent contribution to the overall model fit and permutation importance. Permutation importance represents how heavily the final model depended on a certain variable [29](#_ENREF_29) and was calculated by random values of each variable between presence and background points and then measures resulting drop in AUC with a larger drop indicating higher importance of that variable to the model [30](#_ENREF_30)^,^[31](#_ENREF_31). Then, the receiver operating characteristic (ROC) curve and the area enclosed by the abscissa as the area under the curve (AUC) were used to evaluate model accuracy [24](#_ENREF_24). The AUC value ranges from 0.5 for an uninformative model to 1 for perfect discrimination [32](#_ENREF_32). An AUC value close to 1 indicates more habitat suitability deviation from a random value and a stronger correlation between environmental variables and habitat classification, signifying a better model prediction [33](#_ENREF_33)^,^[34](#_ENREF_34). The suitable habitat maps were calculated using the logistic output of MaxEnt, which ranges from 0 to 1.

The outputs of the MaxEnt model in logistic format were imported into QGIS software for data extraction. China’s map served as the base to extract the potential suitable distribution of *O. microphylla*. The average logical value combined with the actual distribution were used to classify distribution-level values and corresponding distribution ranges. The specific suitability is divided into five categories: fail (0 − 0.15), poor (0.15 − 0.3), fair (0.3 − 0.45), good (0.45 − 0.6), and excellent (>0.6) [35](#_ENREF_35)^,^[36](#_ENREF_36). Different suitable levels indicate the differential potential distribution probability of the species in specific areas. The higher the probability, the more suitable is an area for species occurrence.

3. Results

3.1 Evaluating model performance

The current distribution areas of *O. microphylla* were predicted using the MaxEnt model (Figure 2). The AUC value for the MaxEnt models was 0.962, which was significantly higher than the AUC value of a random prediction (0.5). This result indicated that the prediction results were “excellent”, and the MaxEnt model was reliable for predicting the potential geographical distribution areas of *O. microphylla* in China.

3.2 Key environmental factors and validation of modeling results

The key environmental factors shaping the potential distribution of *O. microphylla* were determined according to their contributions to the MaxEnt modeling process by the jackknife test (Table 2). It found that bio14 (precipitation of the driest month), bio4 (temperature seasonality), and bio16 (precipitation of the wettest quarter) had the highest contribution of 53.3%, 22.6% and 12.2%, respectively (aggregate contribution of 88.1% to the model; Table 2). The mean diurnal range (bio2, 45.8%) and precipitation of the driest month (bio14, 38%) had the highest permutation importance. The correlation coefficients (*r*) between the eight environmental factors were below 0.8. The two bioclimatic factors (bio14, bio2) were identified as the main drivers of the modern geographical distribution of *O. microphylla*.

The results of jackknife tests indicated that when only individual variables were used, the regularized training gain, test gain, and AUC values of the mean diurnal range (bio2) and precipitation of the driest month (bio14) declined the most. These two variables, demonstrating the strongest influence on species distribution, were the main factors influencing the geographical distribution of *O. microphylla* (Figure 3).

The species response curve depicts the relationship between environmental variables and the probability of species incidence. They show the target species’ biological tolerances and habitat preferences. The distribution probability of O. microphylla increased with the value of each environmental variable within a certain range. It decreased with the increase of the variable after reaching a certain peak value (Figure 4). Based on the species response curves, O. microphylla prefers the mean diurnal range (bio2) range from 7 to 8℃, and precipitation of the driest month (bio14) ranges from 30 to 40 mm (Figure 4).

3.3 Predicting suitable habitats of O. microphylla in China

The five categories of *O. microphylla* suitability habitats for the current climate were mapped (Table 3 and Figure 5). The suitable areas for the fail, poor, fair, good, and excellent suitability categories occupied 183.67×10^4^ km^2^, 41.76×10^4^ km^2^, 23.19×10^4^ km^2^, 17.42×10^4^ km^2^, and 16.55×10^4^ km^2^, respectively, comprising 19.13%, 4.35%, 2.42%, 1.81%, and 1.72% of China’s total land area (about 960×10^4^ km^2^) (Table 3). The excellent suitable habitat areas were mainly distributed in the south China provinces of Guangxi, Guizhou, Hunan, Guangdong, and Fujian, covering approximately 14.59×10^4^ km^2^ (Table 3). However, the provinces contiguous or proximal to the main distribution areas, namely Hubei, Zhejiang, and Taiwan, have no distribution records in the good and excellent categories. This pronounced discrepancy indicates that the potential range of *O. microphylla* is considerably larger than the actual distribution range, and the species has the potential to expand the current range to fill the potential areas.

4. Discussion

This is the first study employing the MaxEnt model in conjunction with QGIS to analyze the spatial distribution pattern of *O. microphylla* and climate change impacts on its potential geographical range, and predict its potential distribution area in China quantitatively and intuitively. We built the model with 45 valid occurrence records and eight key environmental factors (Figure 1). The AUC value generated by MaxEnt reached 0.962 (Figure 2), indicating the prediction had a high degree of fit between the climatic variables and the actual suitable habitat of *O. microphylla*. The model jackknife test provided a measure of the contribution of the various environmental variables in influencing the suitability for *O. microphylla*. The prediction results provide a theoretical basis for developing protection and management measures for the endangered species.

This study analyzed the relationship between the occurrence probability of *O. microphylla* and key environmental variables. The results showed that precipitation of the driest month (bio14) was the primary bioclimatic variable affecting the presence of *O. microphylla*, with optimum conditions at 30 to 40 mm. As a plant growth prerequisite, precipitation is the primary limiting factor for almost all species [37](#_ENREF_37)^,^[38](#_ENREF_38). Variations in precipitation and consequent changes in temperature and humidity disrupt the balance of soil moisture and most physiological plant functions [36](#_ENREF_36)^,^[39](#_ENREF_39). The driest month in China occurs in March, April, or May, accompanied by higher temperatures. Moreover, this Spring season is critical for the germination of *O. microphylla* seeds. Sufficient precipitation of the driest month can raise atmospheric humidity and soil moisture to foster seed germination and seedling growth. Nevertheless, little is known about how precipitation affects the survival of *O. microphylla*, and future extensive research could tackle this question.

The MaxEnt model found divergent relative importance for the eight environmental variables. The mean diurnal range (bio2) was the principal temperature variable limiting the potential suitable distribution of *O. microphylla* (Table 2 and Figure 3), with optimum conditions at 7−8 ℃ (Figure 4). The mean diurnal range denotes the temperature variations in a day, which may impact plant growth, especially daytime photosynthesis and respiration that contribute to nutrient buildup [40](#_ENREF_40). Some pertinent processes associated with photosynthesis are highly sensitive to temperature. Low temperatures can reduce the hydrolysis and transport of starch accumulated within the chloroplast to depress the photosynthetic rate. It can also suppress the germination rate and biomass accumulation and modify the survival strategies. On the contrary, the photosynthetic rate increases in response to temperature rise until reaching a thermal optimum, after which the rate declines due to enzyme deactivation at high temperature [41](#_ENREF_41)^,^[42](#_ENREF_42). Other studies have shown faster temperature rises at nighttime than daytime, which is not conducive to nutrient accumulation [43](#_ENREF_43)^,^[44](#_ENREF_44). However, the specific mechanism concerning the influence of mean diurnal range on *O. microphylla* is unclear. Therefore, research on this topic could be strengthened in the future.

The other variables, such as bio4 (temperature seasonality) and bio16 (precipitation of the wettest quarter), have an important influence on the habitat suitability distribution of *O. microphylla* (Table 2). Temperature seasonality represents the temperature variations in a year. A larger standard deviation brings a greater coefficient of variation [45](#_ENREF_45). Therefore, this species is not suitable for an environment with a wide annual temperature amplitude. The wettest period in China occurs in June, July, and August, which also have the highest yearly temperatures [46](#_ENREF_46). Moreover, this Summer season is also a critical period for the maturation of *O. microphylla* fruits and seeds. Sufficient precipitation in the wettest quarter can raise atmospheric humidity and soil moisture to foster fruit growth and maturation. The moist conditions also increase fats and soluble sugars in seeds, boosting the energy supply for germination and initial seedling growth. The ability of seedlings in different habitats to resist harsh environmental conditions will be correspondingly enhanced [47](#_ENREF_47).

Under current climate conditions, the excellent suitable habitats for *O. microphylla* mainly cover Guangxi, Guizhou, Hunan, Guangdong, and Fujian provinces in south China (Table 3 and Figure 5). The model’s prediction matches the existing data, highlighting the accuracy of the prediction. However, the predicted suitable habitat is much larger than the current known species distribution. No occurrence record is found in Sichuan, Yunnan, Hubei, Anhui, Zhejiang, Jiangsu, and Taiwan provinces, even though they are the species’ potentially important habitats. This notable discrepancy may be related to limited research on this species, resulting in inadequate data to describe species environmental requirements accurately. On the other hand, MaxEnt has the inherent trait of evaluating only niche-based species presence data [48](#_ENREF_48). The model predicts the species’ fundamental niche rather than the actual niche, resulting in the predicted potential distribution area being larger than the actual range. However, the species may fail to disperse to some suitable areas due to biogeographical barriers, such as human interference, topographical obstacles, and inter-specific competition [49](#_ENREF_49).

Based on the predicted results, the suitable habitat of *O. microphylla* is relatively widespread. However, in reality, the prospect of population development is not optimistic. To prevent the extinction of this endangered plant, in situ conservation of the known *O. microphylla* community is necessary to minimize the adverse effects of human activities on species survival. More distribution point information can be obtained by conducting *O. microphylla* studies to deepen the knowledge of biological characteristics, genetic structure, and artificial cultivation methods, as well as investigating and monitoring the apparent “omission” areas. Collecting seeds for artificial breeding and selecting potential suitable areas with less human activity for wild release can expand the spread and size of wild populations.

The ability of the MaxEnt model to use scarce distribution records to infer the environmental tolerance and ecological niches of *O. microphylla* is supported by the generation of highly accurate predictions. However, it was difficult for the species to establish and survive in all suitable areas. The distribution areas of its communities have been progressively reduced despite the expansion of suitable habitats [50](#_ENREF_50). Besides external environmental and climatic conditions, other factors may have important implications for species distribution at different spatial scales [51](#_ENREF_51). They include physiological constraints, topography, soil, human disturbance, spatial constraint, dispersal mode, competition, and response to external factors and drivers. For instance, the seeds of *O. microphylla* are hard and compact, with a less permeable cortex and a long dormancy period. Thus, its germination rate is extremely low, making it difficult to survive in suitable areas [21](#_ENREF_21). In addition, land use and land cover changes caused by human activities have significantly impacted species distribution [52](#_ENREF_52). However, these factors take time to take effect, which may cause species distribution to lag behind climate change [53](#_ENREF_53).

Under the climatic conditions of increasing CO_2_ concentration and temperature in the future, the annual mean precipitation may increase across China [54](#_ENREF_54). Our research showed that the main factors influencing the distribution of *O. microphylla* were the mean diurnal range and precipitation of the driest month. With the increase of temperature and precipitation in the future climate change, sufficient soil moisture and suitable soil temperature can better meet the needs of seed germination and vegetation growth [55](#_ENREF_55). Therefore, the suitable area of *O. microphylla* may vary with the magnitude of future climate change.

In short, predicted results based on the MaxEnt model could help identify additional localities where the *O. microphylla* may already exist but have not yet been detected. Alternatively, the predicted suitable areas offer new habitats to expand the species range or are used as priority areas for introducing and cultivating this rare tree. Therefore, deeper cognate research should be conducted to expand the knowledge and practice base. The locations of key protected areas for *O. microphylla* could be identified to strengthen their supervision and management and minimize human activities. Uninformed and subjective designation of protection areas can be avoided. The numerical range of the main environmental variables can provide an objective reference to select artificial breeding and rescue sites. Thus, the protected plants can be nurtured in appropriate environmental conditions to rationalize resource development and utilization. Moreover, this study indicates that other aspects can be improved. In addition to the selected bioclimatic variables, other factors such as soil, water, land use change, different climate scenarios, and dispersal ability may affect the prediction results. Therefore, future studies can comprehensively consider a broader range of environmental factors to develop a response strategy for *O. microphylla* to tackle climate change impacts.

5. Conclusion

The suitable areas of *O. microphylla* in China have been accurately predicted by MaxEnt modeling using mainly bioclimatic variables. The excellent and good suitable habitats were found primarily in China’s southern provinces, including Guizhou, Hunan, Guangxi, Guangdong, and Fujian provinces. The crucial environmental variables regulating its distribution are the precipitation of the driest month (bio14) and mean diurnal range (bio2), with the optimal conditions at 30−40 mm and 7−8 ℃, respectively. The results could pinpoint the specific conditions and locations for the optimal growth of the species and provide the scientific basis to improve the management and conservation measures for this endangered species.

**Data Availability Statement**

The data presented in this study are available on request from the first author.

**References**

1 Sax, D. F., Early, R. & Bellemare, J. Niche syndromes, species extinction risks, and management under climate change. *Trends in Ecology & Evolution* **28**, 517-523, doi:10.1016/j.tree.2013.05.010 (2013).

2 Ceballos, G. *et al.* Accelerated modern human–induced species losses: Entering the sixth mass extinction. *Science Advances* **1**, e1400253, doi:10.1126/sciadv.1400253 (2015).

3 Kumar, D., Rawat, S. & Joshi, R. Predicting the current and future suitable habitat distribution of the medicinal tree *Oroxylum indicum* (L.) Kurz in India. *Journal of Applied Research on Medicinal and Aromatic Plants* **23**, 100309, doi:<https://doi.org/10.1016/j.jarmap.2021.100309> (2021).

4 Abolmaali, S. M.-R., Tarkesh, M. & Bashari, H. MaxEnt modeling for predicting suitable habitats and identifying the effects of climate change on a threatened species, *Daphne mucronata*, in central Iran. *Ecological Informatics* **43**, 116-123, doi:<https://doi.org/10.1016/j.ecoinf.2017.10.002> (2018).

5 Wang, G. *et al.* Predicting suitable habitats for china's endangered plant *Handeliodendron bodinieri* (H. L&eacute;v.) Rehder. *Diversity* **15**, 1033, doi:10.3390/d15101033 (2023).

6 Franklin, J. Species distribution models in conservation biogeography: developments and challenges. *Diversity and Distributions* **19**, 1217-1223, doi:<https://doi.org/10.1111/ddi.12125> (2013).

7 Nguyen, T. T., Gliottone, I. & Pham, M. P. Current and future predicting habitat suitability map of *Cunninghamia konishii* Hayata using MaxEnt model under climate change in Northern Vietnam. *European Journal of Ecology* **7**, doi:10.17161/eurojecol.v7i2.15079 (2021).

8 Graham, E. M. *et al.* Climate change and biodiversity in Australia: a systematic modelling approach to nationwide species distributions. *Australasian Journal of Environmental Management* **26**, 112-123, doi:10.1080/14486563.2019.1599742 (2019).

9 Mousavi Kouhi, S. M. & Erfanian, M. Predicting the present and future distribution of medusahead and barbed goatgrass in Iran. *ECOPERSIA* **8**, 41-46 (2020).

10 Dudley, T. L. & Bean, D. W. *Tamarisk biocontrol*, endangered species risk and resolution of conflict through riparian restoration. *BioControl* **57**, 331-347, doi:10.1007/s10526-011-9436-9 (2012).

11 Wilson, J. W., Sexton, J. O., Todd Jobe, R. & Haddad, N. M. The relative contribution of terrain, land cover, and vegetation structure indices to species distribution models. *Biological Conservation* **164**, 170-176, doi:<https://doi.org/10.1016/j.biocon.2013.04.021> (2013).

12 Elith, J. & Leathwick, J. R. Species distribution models: ecological explanation and prediction across space and time. **40**, 677-697, doi:<https://doi.org/10.1146/annurev.ecolsys.110308.120159> (2009).

13 Rovzar, C., Gillespie, T. W. & Kawelo, K. Landscape to site variations in species distribution models for endangered plants. *Forest Ecology and Management* **369**, 20-28, doi:<https://doi.org/10.1016/j.foreco.2016.03.030> (2016).

14 Miller, J. Species Distribution Modeling. **4**, 490-509, doi:<https://doi.org/10.1111/j.1749-8198.2010.00351.x> (2010).

15 Gelviz-Gelvez, S. M., Pavón, N. P., Illoldi-Rangel, P. & Ballesteros-Barrera, C. Ecological niche modeling under climate change to select shrubs for ecological restoration in Central Mexico. *Ecological Engineering* **74**, 302-309, doi:<https://doi.org/10.1016/j.ecoleng.2014.09.082> (2015).

16 Elith, J. *et al.* A statistical explanation of MaxEnt for ecologists. **17**, 43-57, doi:<https://doi.org/10.1111/j.1472-4642.2010.00725.x> (2011).

17 Cai, C., Zhang, X., Zha, J., Li, J. & Li, J. Predicting climate change impacts on the rare and endangered *Horsfieldia tetratepala* in China. *Forests* **13**, 1051, doi:10.3390/f13071051 (2022).

18 Arshad, F. *et al.* Predicting the suitable current and future potential distribution of the native endangered tree *Tecomella undulata* (Sm.) Seem. in Pakistan. *Sustainability* **14**, 7215, doi:10.3390/su14127215 (2022).

19 Tulloch, A. I. T. *et al.* Conservation planners tend to ignore improved accuracy of modelled species distributions to focus on multiple threats and ecological processes. *Biological Conservation* **199**, 157-171, doi:<https://doi.org/10.1016/j.biocon.2016.04.023> (2016).

20 Tang, J., Zou, R., Wei, X. & Li, D. Complete chloroplast genome sequences of five *Ormosia* Species: Molecular structure, comparative analysis, and phylogenetic analysis. *Horticulturae* **9**, 796, doi:10.3390/horticulturae9070796 (2023).

21 Yang, H., Feng, B., Yuan, M. & Yang, J. Analyses on population pattern and endangered causes of rare and endangered species *Ormosia micropylla*. *Seed* **32**, 52-54, doi:10.3969/j.issn.1001-4705.2013.06.013 (2013).

22 Feng, B., Wang, D., Yang, J. & Wu, M. Study on population composition and structure of *Ormosia micropylla* located in Nanshao, Jianhe County. *Seed* **31**, 65-68, doi:10.3969/j.issn.1001-4705.2012.04.019 (2012).

23 Puchałka, R. *et al.* Black locust (*Robinia pseudoacacia* L.) range contraction and expansion in Europe under changing climate. *Global Change Biology* **27**, 1587-1600, doi:<https://doi.org/10.1111/gcb.15486> (2021).

24 Liu, D., Xie, C., Jim, C. Y., Liu, Y. & Hou, S. Predicting the potential distribution of the alien invasive alligator gar *Atractosteus spatula* in China. *Sustainability* **15**, 6419, doi:10.3390/su15086419 (2023).

25 Rana, S. K., Rana, H. K., Ghimire, S. K., Shrestha, K. K. & Ranjitkar, S. Predicting the impact of climate change on the distribution of two threatened Himalayan medicinal plants of Liliaceae in Nepal. *Journal of Mountain Science* **14**, 558-570, doi:10.1007/s11629-015-3822-1 (2017).

26 Muscarella, R. *et al.* ENMeval: An R package for conducting spatially independent evaluations and estimating optimal model complexity for Maxent ecological niche models. **5**, 1198-1205, doi:<https://doi.org/10.1111/2041-210X.12261> (2014).

27 Phillips, S. J., Anderson, R. P., Dudík, M., Schapire, R. E. & Blair, M. E. Opening the black box: An open-source release of Maxent. **40**, 887-893, doi:<https://doi.org/10.1111/ecog.03049> (2017).

28 Wang, X. *et al.* Prediction of historical, present and future distribution of *Quercus* sect. *Heterobalanus* based on the optimized MaxEnt model in China. *Acta Ecologica Sinica* **43**, 6590-6604, doi:10.5846/stxb202205141353 (2023).

29 Howse, M. W. F., Haywood, J. & Lester, P. J. Bioclimatic modelling identifies suitable habitat for the establishment of the invasive european paper wasp (Hymenoptera: Vespidae) across the Southern Hemisphere. *Insects* **11**, 784, doi:10.3390/insects11110784 (2020).

30 Préau, C., Trochet, A., Bertrand, R., R. & Isselin-Nondedeu, F. Modeling potential distributions of three European amphibian species comparing ENFA and MaxEnt. *Amphibia-Reptilia* **13**, 91–104 (2018).

31 Swan, M., Le Pla, M., Di Stefano, J., Pascoe, J. & Penman, T. D. Species distribution models for conservation planning in fire‐prone landscapes. *Biodiversity and Conservation* **30**, 1119-1136, doi:10.1007/s10531-021-02136-4 (2021).

32 Gastón, A. & García-Viñas, J. I. Modelling species distributions with penalised logistic regressions: A comparison with maximum entropy models. *Ecological Modelling* **222**, 2037-2041, doi:<https://doi.org/10.1016/j.ecolmodel.2011.04.015> (2011).

33 Elith, J. *et al.* Novel methods improve prediction of species’ distributions from occurrence data. *Ecography* **29**, 129-151, doi:<https://doi.org/10.1111/j.2006.0906-7590.04596.x> (2006).

34 Ye, X.-z. *et al.* Distribution pattern of endangered plant *Semiliquidambar cathayensis* (Hamamelidaceae) in response to climate change after the last interglacial period. *Forests* **11**, 434, doi:10.3390/f11040434 (2020).

35 Xie, C., Huang, B., Jim, C. Y., Han, W. & Liu, D. Predicting differential habitat suitability of *Rhodomyrtus tomentosa* under current and future climate scenarios in China. *Forest Ecology and Management* **501**, 119696 (2021).

36 Sarma, K. *et al.* Habitat suitability of Gymnocladus assamicus - A critically endangered plant of Arunachal Pradesh, India using machine learning and statistical modeling. *Acta Ecologica Sinica* **42**, 398-406, doi:<https://doi.org/10.1016/j.chnaes.2022.05.009> (2022).

37 Marcer, A., Sáez, L., Molowny-Horas, R., Pons, X. & Pino, J. Using species distribution modelling to disentangle realised versus potential distributions for rare species conservation. *Biological Conservation* **166**, 221-230, doi:<https://doi.org/10.1016/j.biocon.2013.07.001> (2013).

38 Ngarega, B. K., Masocha, V. F. & Schneider, H. Forecasting the effects of bioclimatic characteristics and climate change on the potential distribution of *Colophospermum mopane* in southern Africa using Maximum Entropy (Maxent). *Ecological Informatics* **65**, 101419, doi:<https://doi.org/10.1016/j.ecoinf.2021.101419> (2021).

39 Xing, Y. *et al.* The current distribution of *Carex alatauensis* in the Qinghai-Tibet Plateau estimated by MaxEnt. *Agronomy* **13**, 564, doi:10.3390/agronomy13020564 (2023).

40 Nzei, J. M. *et al.* Assessment of climate change and land use effects on water lily (Nymphaea L.) habitat suitability in South America. *Diversity* **14**, 830, doi:10.3390/d14100830 (2022).

41 Prasad, P. V. V. & Djanaguiraman, M. High night temperature decreases leaf photosynthesis and pollen function in grain sorghum. *Functional Plant Biology* **38**, 993-1003 (2011).

42 Tao, Z. Predicting the changes in suitable habitats for six common woody species in Central Asia. *International Journal of Biometeorology* **67**, 107-119, doi:10.1007/s00484-022-02389-w (2023).

43 Cheng, R. *et al.* Predicting the potential suitable distribution of *Larix principis-rupprechtii* Mayr under climate change scenarios. *Forests* **13**, 1428, doi:10.3390/f13091428 (2022).

44 Wang, X. *et al.* Modeling the potential distribution of three taxa of *Akebia* Decne. under climate change scenarios in China. *Forests* **12**, 1710, doi:10.3390/f12121710 (2021).

45 Liu, Y. *et al.* MaxEnt modelling for predicting the potential distribution of a near threatened rosewood species (*Dalbergia cultrata* Graham ex Benth). *Ecological Engineering* **141**, 105612, doi:<https://doi.org/10.1016/j.ecoleng.2019.105612> (2019).

46 Fu, L. *et al.* Integrating regional climate change into allometric equations for estimating tree aboveground biomass of Masson pine in China. *Annals of Forest Science* **74**, 42, doi:10.1007/s13595-017-0636-z (2017).

47 Ma, B. & Sun, J. Predicting the distribution of *Stipa purpurea* across the Tibetan Plateau via the MaxEnt model. *BMC Ecology* **18**, 10, doi:10.1186/s12898-018-0165-0 (2018).

48 Wang, G. *et al.* Integrating Maxent model and landscape ecology theory for studying spatiotemporal dynamics of habitat: Suggestions for conservation of endangered Red-crowned crane. *Ecological Indicators* **116**, 106472, doi:<https://doi.org/10.1016/j.ecolind.2020.106472> (2020).

49 Zhou, Y., Lu, X. & Zhang, G. Potentially differential impacts on niche overlap between Chinese endangered *Zelkova schneideriana* and its associated tree species under climate change. *Frontiers in Ecology and Evolution* **11**, 1218149, doi:10.3389/fevo.2023.1218149 (2023).

50 Qun, L. H., Gang, X. L. & Ping, S. X. Predicting the potential distribution of *Taxus wallichiana* var *mairei* under climate change in china using maxent modeling. *Pakistan Journal of Botany* **54**, 1305-1310, doi:<http://dx.doi.org/10.30848/PJB2022-4(9>) (2022).

51 Eiserhardt, W. L., Svenning, J.-C., Kissling, W. D. & Balslev, H. Geographical ecology of the palms (Arecaceae): determinants of diversity and distributions across spatial scales. *Annals of Botany* **108**, 1391-1416, doi:10.1093/aob/mcr146 (2011).

52 Piao, S. *et al.* Detection and attribution of vegetation greening trend in China over the last 30 years. *Global Change Biology* **21**, 1601-1609, doi:<https://doi.org/10.1111/gcb.12795> (2015).

53 Zhang, L., Jing, Z., Li, Z., Liu, Y. & Fang, S. Predictive Modeling of suitable habitats for *Cinnamomum Camphora* (L.) Presl using maxent model under climate change in China. *International Journal of Environmental Research and Public Health* **16**, 3185, doi:10.3390/ijerph16173185 (2019).

54 Tian, J. *et al.* Projections of precipitation over China based on CMIP6 models. *Stochastic Environmental Research and Risk Assessment* **35**, 831-848, doi:10.1007/s00477-020-01948-0 (2021).

55 Wang, M. & Guan, Q. Prediction of potential suitable areas for *Broussonetia papyrifera* in China using the MaxEnt model and CIMP6 data. *Journal of Plant Ecology* **16**, rtad006, doi:10.1093/jpe/rtad006 (2023).

**Author contributions**

W. L., W. G., Jim C. Y., X.C.: conception, software, methodology, original draft writing. X.C., Jim C. Y.: review and editing. W. L., W. G., G. Z., H. Q.: investigation, data resource management, visualization. X.C., W. G.: supervision, review and financial support. W. L. and W. G. contributed equally to this work and shared the first authorship. All authors contributed to the article and approved the submitted version.

**Funding**

This research was supported by grants awarded by the Guangxi Natural Science Foundation (No.2023GXNSFAA026422); the Basic Ability Enhancement Program for Young and Middle-aged Teachers of Guangxi (No.2024KY0782); the Scientific Research Foundation of Guangxi MinZu Normal University (No.2021BS002; No.2022YB032).

**Competing interests**

The authors declare no competing interests.

**List of figures and tables**

**Figure 1.** The locations of 45 verified current occurrence points of *O. microphylla* (white circles) in central and south China with reference to elevation. The map was prepared by Lijuan Wei, Guohai Wang and Chunping Xie in QGIS 3.34.0 (https://www.qgis.org/en/site/).

**Figure 2**. The validation of the MaxEnt model predicting *O. microphylla* distribution: (a) Omission rate; and (b) ROC curve.

**Figure 3.** The jackknife test for evaluating the relative importance of environmental variables for *O. microphylla*.

**Figure 4.** Response curves of the environmental variables to distribution probability.

**Figure 5**. The predicted *O. microphylla* distribution range classified into five suitability categories obtained by MaxEnt modeling. The map was prepared by Lijuan Wei, Guohai Wang and Chunping Xie in QGIS 3.34.0 (https://www.qgis.org/en/site/).

**Table 1**. List of 19 environmental variables considered at the initial stage of model development. Eight variables with the code shown in bold font were chosen for the MaxEnt modeling study.

**Table 2**. Percent contribution and permutation importance levels of the eight environmental variables included in the MaxEnt models, ranked by percentage contribution.

**Table 3** Predicted suitable areas for *O. microphylla* under the current climate scenario in various provinces (10^4^ km^2^). The ratio denotes the predicted suitable area divided by the total land area of the respective province or autonomous.

**Figure 1.** The locations of 45 verified current occurrence points of *O. microphylla* (white circles) in central and south China with reference to elevation. The map was prepared by Lijuan Wei, Guohai Wang and Chunping Xie in QGIS 3.34.0 (https://www.qgis.org/en/site/).

**Figure 2**. The validation of the MaxEnt model predicting *O. microphylla* distribution: (a) Omission rate; and (b) ROC curve.


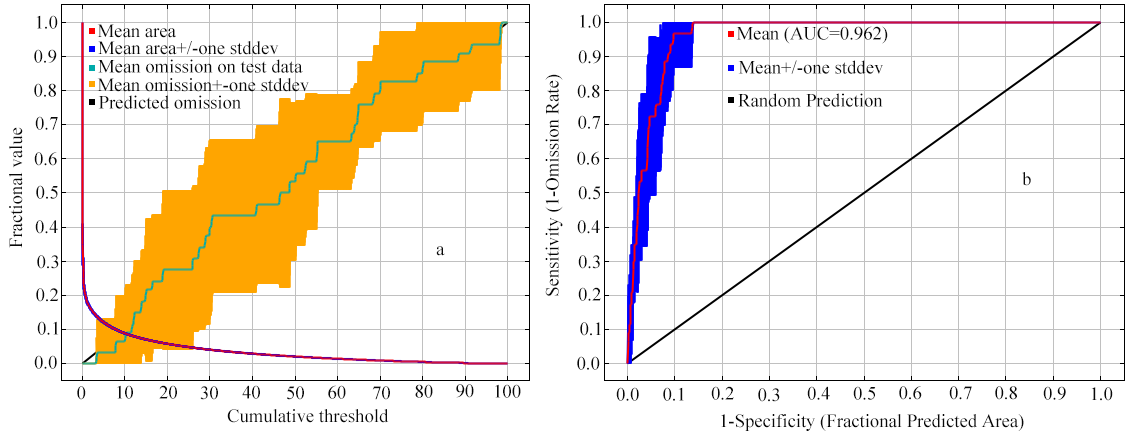


**Figure 3.** The jackknife test for evaluating the relative importance of environmental variables for *O. microphylla*.


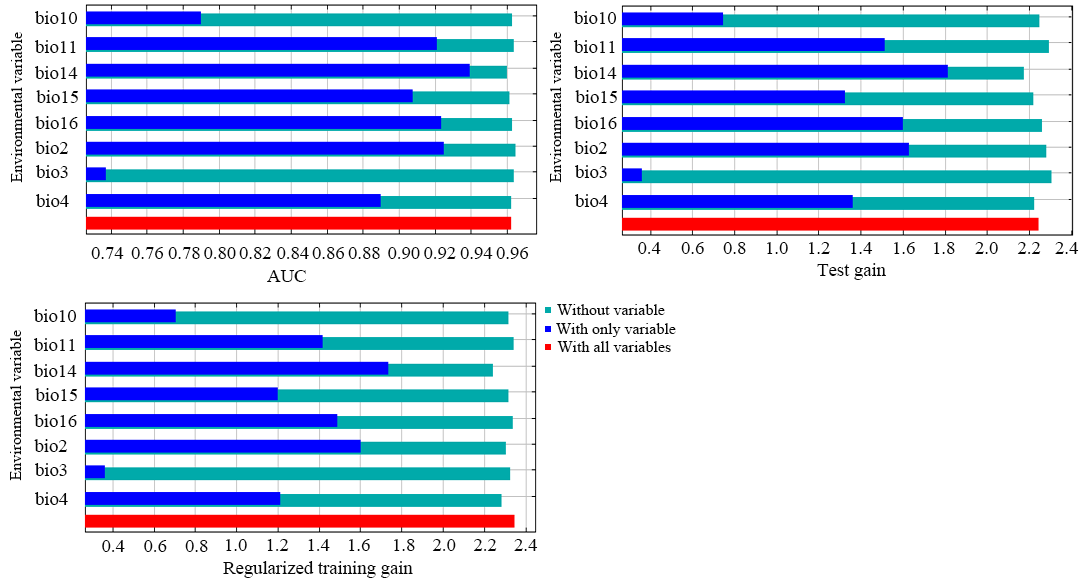


**Figure 4.** Response curves of the environmental variables to distribution probability.


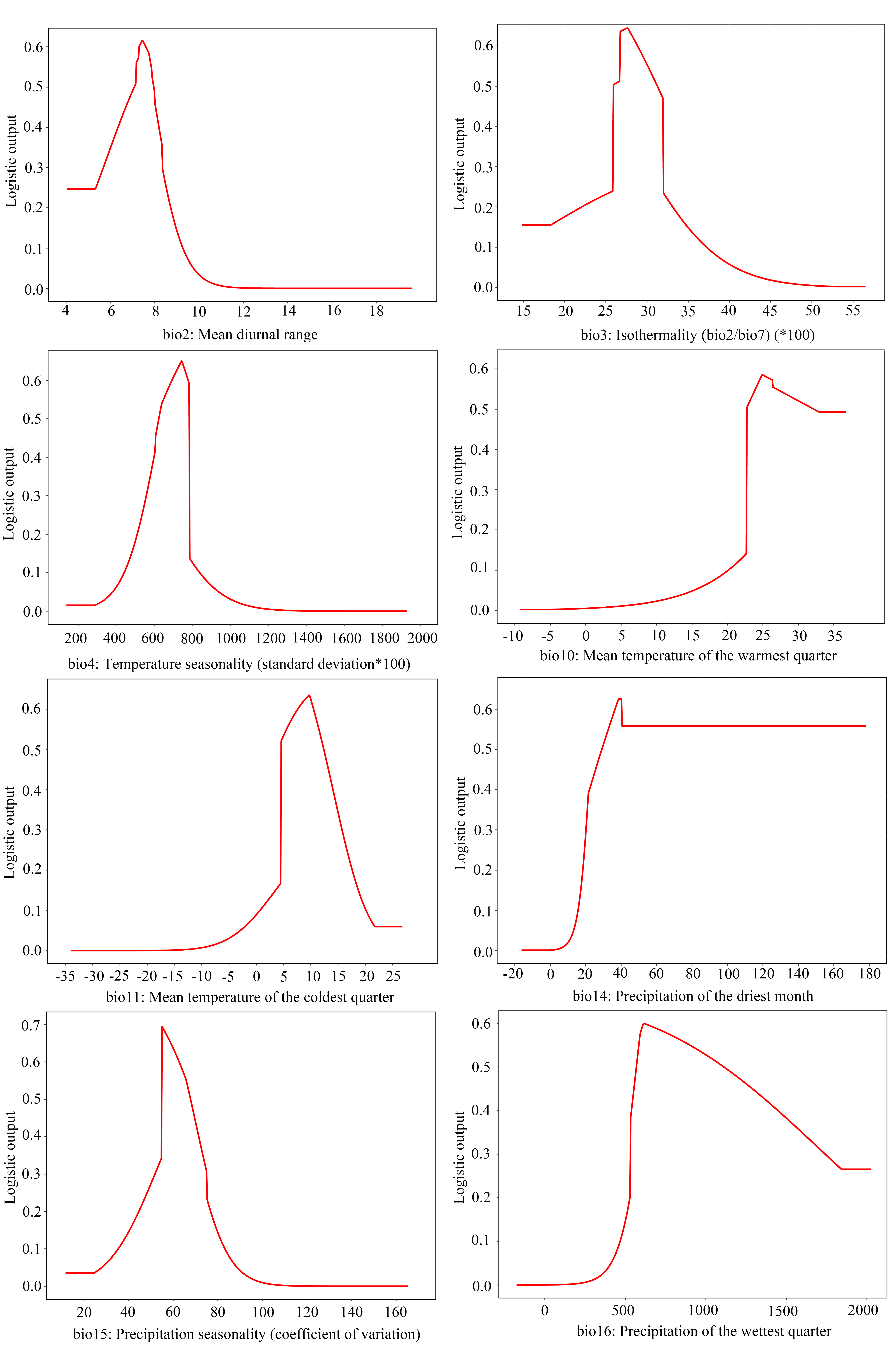


**Figure 5**. The predicted *O. microphylla* distribution range classified into five suitability categories obtained by MaxEnt modeling. The map was prepared by Lijuan Wei, Guohai Wang and Chunping Xie in QGIS 3.34.0 (https://www.qgis.org/en/site/).


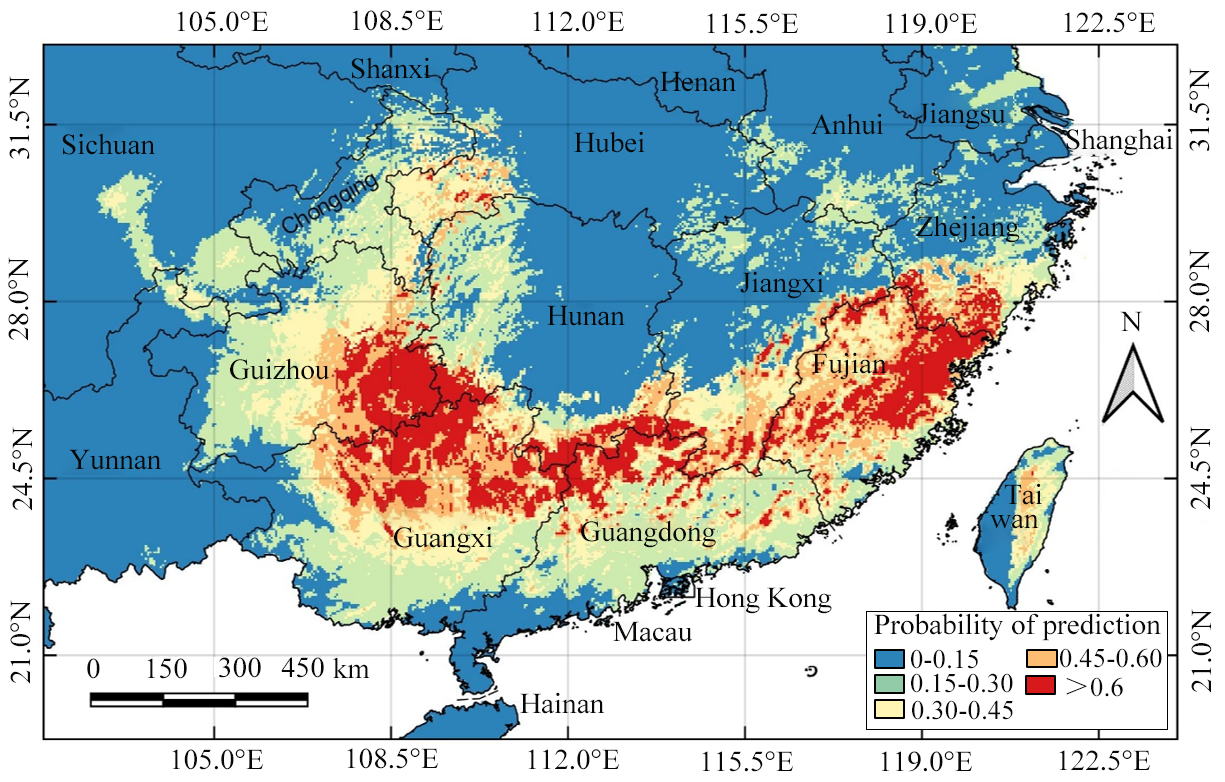


**Table 1**. List of 19 environmental variables considered at the initial stage of model development. Eight variables with the code shown in bold font were chosen for the MaxEnt modeling study.

| **Code** | **Environmental variable** | **Unit** |
| --- | --- | --- |
| bio1 | Annual mean temperature | ℃ |
| **bio2** | **Mean diurnal range (mean of monthly (max temp-min temp))** | ℃ |
| **bio3** | **Isothermality (bio2/bio7) (*100)** | - |
| **bio4** | **Temperature seasonality (standard deviation*100)** | ℃ |
| bio5 | Max temperature of the warmest month | ℃ |
| bio6 | Min temperature of the coldest month | ℃ |
| bio7 | Temperature annual range (bio5-bio6) | ℃ |
| bio8 | Mean temperature of the wettest quarter | ℃ |
| bio9 | Mean temperature of the driest quarter | ℃ |
| **bio10** | **Mean temperature of the warmest quarter** | ℃ |
| **bio11** | **Mean temperature of the coldest quarter** | ℃ |
| bio12 | Annual precipitation | mm |
| bio13 | Precipitation of the wettest month | mm |
| **bio14** | **Precipitation of the driest month** | mm |
| **bio15** | **Precipitation seasonality (coefficient of variation)** | - |
| **bio16** | **Precipitation of the wettest quarter** | mm |
| bio17 | Precipitation of the driest quarter | mm |
| bio18 | Precipitation of the warmest quarter | mm |
| bio19 | Precipitation of the coldest quarter | mm |

**Table 2**. Percent contribution and permutation importance levels of the eight environmental variables included in the MaxEnt models, ranked by percentage contribution.

| **Code** | **Bioclimatic variable** | **Percent contribution (%)** | **Permutation importance (%)** |
| --- | --- | --- | --- |
| bio14 | Precipitation of the driest month | 53.3 | 38.0 |
| bio4 | Temperature seasonality | 22.6 | 6.8 |
| bio16 | Precipitation of the wettest quarte | 12.2 | 1.0 |
| bio2 | Mean diurnal range | 3.7 | 45.8 |
| bio10 | Mean temperature of the warmest quarter | 2.9 | 2.1 |
| bio3 | Isothermality (Bio2/Bio7) (*100) | 2.2 | 2.5 |
| bio11 | Mean temperature of the coldest quarter | 2.0 | 1.2 |
| bio15 | Precipitation seasonality | 1.1 | 2.6 |

**Table 3** Predicted suitable areas for *O. microphylla* under the current climate scenario in various provinces (10^4^ km^2^). The ratio denotes the predicted suitable area divided by the total land area of the respective province or autonomous.

| **Province** | **Probability of prediction** | | | | |
| --- | --- | --- | --- | --- | --- |
|  | Fail  (0-0.15) | Poor  (0.15-0.3) | Fair  (0.3-0.45) | Good (0.45-0.6) | Excellent  (>0.6) |
| Guangxi | 4.06 | 6.09 | 4.68 | 4.47 | 4.46 |
| Fujian | 0.18 | 1.41 | 2.82 | 3.88 | 3.86 |
| Guizhou | 3.12 | 5.55 | 3.90 | 2.30 | 2.75 |
| Guangdong | 3.67 | 6.91 | 3.72 | 1.68 | 1.98 |
| Hunan | 12.58 | 4.35 | 1.58 | 1.14 | 1.54 |
| Jiangxi | 9.16 | 2.73 | 2.01 | 1.84 | 0.95 |
| Zhejiang | 4.77 | 2.42 | 1.10 | 0.99 | 0.89 |
| Hubei | 15.06 | 1.35 | 1.48 | 0.60 | 0.10 |
| Taiwan | 1.62 | 0.90 | 0.72 | 0.36 | 0.01 |
| Chongqing | 3.48 | 3.39 | 1.22 | 0.16 | 0.01 |
| Sichuan | 45.24 | 2.96 | 0.40 | 0.00 | 0.00 |
| Yunnan | 38.7 | 0.57 | 0.14 | 0.00 | 0.00 |
| Anhui | 12.11 | 1.77 | 0.13 | 0.00 | 0.00 |
| Jiangsu | 9.45 | 1.27 | 0.00 | 0.00 | 0.00 |
| Shanxi | 20.47 | 0.09 | 0.00 | 0.00 | 0.00 |
| Total | 183.67 | 41.76 | 23.19 | 17.42 | 16.55 |
